# Emergency medical services in low- and middle-income countries: a systematic review of systemic barriers and integration challenges

**DOI:** 10.1016/j.pmedr.2026.103522

**Published:** 2026-06-03

**Authors:** Alina Pant, Thunwadee Tachapattaworakul Suksaroj, Cheerawit Rattanapan, Orapin Laosee, Piyapong Janmaimool, Kwang Mo Yang

**Affiliations:** a Graduate Program of Health and Sustainable Development, Mahidol University, Nakhon Pathom, Thailand; b ASEAN Institute for Health Development, Mahidol University, Nakhon Pathom, Thailand; c Center for Global Health, Mahidol University, Nakhon Pathom, Thailand

**Keywords:** Emergency medical services, Prehospital care, Low- and middle-income countries, Health systems

## Abstract

**Objective:**

To assess the status of emergency medical services (EMS) in low- and middle-income countries (LMICs) and identify key barriers to effective service delivery.

**Methods:**

A systematic review was conducted following PRISMA guidelines. PubMed, Scopus, Embase, and Cochrane Library were searched in March 2025 for studies on emergency medical service systems in low- and middle-income countries published between 2009 and 2023. Seventeen peer-reviewed studies were included. Thematic analysis was applied to synthesize findings and identify recurring challenges across EMS systems.

**Results:**

EMS systems in LMICs ranged from fragmented and informal arrangements to partially integrated national systems. Key barriers included weak governance, inadequate financing, workforce shortages, poor communication and coordination, and limited infrastructure. Findings suggest that EMS underperformance is driven not only by resource constraints but also by fragmentation and limited integration of community-based emergency networks. Informal responders, such as community volunteers, frequently fill gaps in formal EMS but remain largely absent from policy frameworks.

**Conclusions:**

Strengthening EMS in LMICs requires integrated approaches that bridge formal and informal systems, alongside improved workforce capacity and coordination, to enhance emergency care outcomes.

## Highlights

• Emergency care challenges extend beyond resource limitations.
• Fragmented systems and weak governance reduce service effectiveness.
• Informal responders play a vital but underrecognized role.
• Funding shifts may affect emergency care sustainability.
• Integrated local models can strengthen emergency care systems.

## 1 Introduction

An emergency medical service (EMS) system is a coordinated network of personnel, facilities, and equipment that provides effective and timely delivery of health and safety services during medical emergencies (Mehmood et al., 2018), to reduce preventable mortality and long-term morbidity (Abuzeyad et al., 2020). The core functions of the EMS can be simplified into four main components, which are: (1) access to emergency care, (2) care provided to the community, (3) care on the way to the hospital, and (4) care upon arrival at the receiving facility (Al-Shaqsi, 2010). EMS is a critical component of the healthcare system that provides prehospital care for a wide range of medical conditions (Kumar et al., 2008).

Despite its importance, evidence on EMS systems remains disproportionately concentrated in high-income countries (HICs) (Gage et al., 2023), with comparatively limited representation from LMIC contexts, creating a significant knowledge gap on EMS in low- and middle-income countries (LMICs). In many low- and middle-income countries, formal emergency medical services are often limited or underdeveloped. As a result, informal and community-based emergency response systems, including the use of private transport, community volunteers, and non-specialized providers, play a significant role in prehospital care. These systems, while essential for access, are frequently fragmented and lack integration with formal health systems [S6, S12].

In LMICs, the majority of premature deaths from emergency conditions are due to inadequate out-of-hospital care or a poor transport system (Mehmood et al., 2018). Key barriers include scarcity of trained EMS personnel, limited availability of essential medicine and equipment, and delayed ambulance times [S12]. Prehospital care can reduce trauma-related mortality and fatal injuries by more than 25% and significantly decrease disability-adjusted life years (DALYs) (Mehmood et al., 2018; WHO, 2005). Almost 80% of trauma-related deaths in LMICs are attributed to the absence of adequate prehospital emergency care [S2]. For instance, Lao PDR lacks an emergency triage system and relies on non-standardized assessment tools, which limits the effectiveness of early emergency response (Silavong et al., 2023). Similarly, Nepal suffers from uncoordinated and unintegrated EMS systems, due to a lack of standardized ambulance service and low public awareness and accessibility (Gongal et al., 2009). This partly explains the significant need to address barriers to EMS systems in LMICs.

Poor infrastructure and a non-standardized EMS system are particularly vulnerable during a disaster situation (Al Harthi et al., 2020). With increasing threats of climate change leading to increased frequency and severity of natural disasters such as wildfires, floods, and heatwaves, the need for effective EMS systems in LMICs is more urgent than ever (Rocha et al., 2022; Rentschler et al., 2022). Although previous systematic reviews have described EMS challenges in LMICs [S2, S12], the current EMS landscape is changing rapidly in LMICs.

Importantly, much of the existing literature assumes that EMS development in LMICs should follow models established in high-income countries (Moussally et al., 2024). However, this assumption may not be appropriate, as LMICs often operate within fundamentally different health system structures, resource constraints, and social contexts (Moussally et al., 2024). Understanding how existing formal and informal systems interact is therefore essential for developing context-appropriate EMS models.

Therefore, this systematic review seeks to describe the current EMS situation in LMICs and analyze systemic and policy-level barriers that obstruct the integration and sustainability of EMS within national health systems. Specifically, the review aims to address the following research questions, framed within broader health system and policy discourse:

1. What is the current status of the EMS system in LMICs?
2. What systematic and policy barriers limit the integration of EMS into national health systems?

## 2 Methods

### 2.1 Protocol

The protocol for this systematic review has been published in the International Prospective Register of Systematic Reviews (PROSPERO) database (registration ID: CRD42023480845). The review was conducted according to Preferred Reporting Items for Systematic reviews and Meta-Analyses (PRISMA) guidelines. While the original protocol specified a content analysis, the synthesis process revealed that the data required iterative coding, theme development, and the interpretation of patterned meaning.

### 2.2 Search strategy and screening

Articles published on the emergency medical service systems and prehospital care were systematically reviewed. The review focused on prehospital emergency services rather than the in-hospital clinical aspects. Four electronic databases were used for this systematic review, namely, PubMed, Scopus, Embase, and Cochrane Library. These databases were selected based on their breadth and depth of indexing documents. The search string incorporated Boolean operators combining key search terms related to EMS: (“ambulance services” OR “emergency” OR “barriers to prehospital care” OR “current status” OR “development” OR “emergency care” OR “emergency medical services” OR “EMS system” OR “out of hospital” OR “prehospital care”) AND (“low- and middle-income countries”). The terms “paramedic” and “paramedicine” were not included in the search strategy because the focus of this review was on system-level EMS structures and integration rather than profession-specific or clinical training-related studies. Searches were conducted in March 2025.

### 2.3 Criteria for study selection

Our search across multiple databases identified a total of 33,435 records published between 2009 and 2023. After removing the duplicates, non-English articles, and non-peer-reviewed articles, 25,384 records remained. Two reviewers (KMY and AP) independently screened titles and abstracts, excluding 25,244 records based on the criteria in Table 1.

**Table 1:** Inclusion and exclusion criteria used for selecting studies on emergency medical service systems in low- and middle-income countries published between 2009 and 2023.

Category	Inclusion criteria	Exclusion Criteria
Publication type	Peer-reviewed journal articles, review articles, mixed-method studies, cross-sectional descriptive studies, and survey reports.	Others.
Publication date	Publications published from 2009 to 2023.	Publications before 2009 and after 2023.
Language	English publications.	Non-English publications.
EMS-related topics	Publications related to the EMS system-related issues.	Publications not connected to EMS systems.
Availability	Full text available.	Full text not available.

**Fig. 1:**
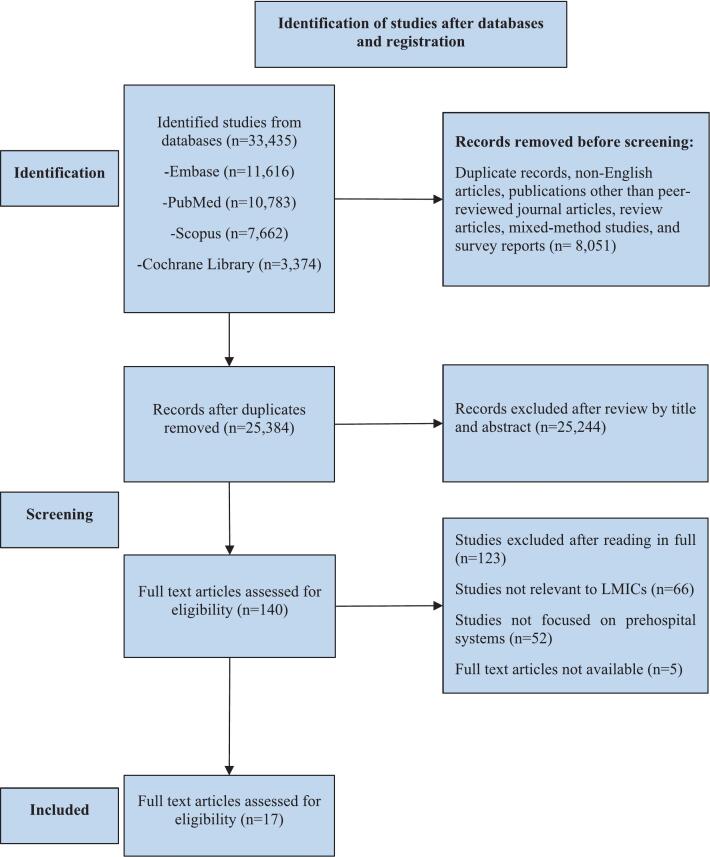
Preferred Reporting Items for Systematic Reviews and Meta-Analyses (PRISMA) flow diagram showing the identification, screening, eligibility assessment, and inclusion of studies evaluating emergency medical service systems in low- and middle-income countries published between 2009 and 2023.

Of the remaining 140 articles selected for full-text assessment, 123 were excluded. Reasons for exclusion at the full-text stage were categorized and documented, including study scope, population, and relevance to EMS system-level analysis, and are summarized in the PRISMA flow diagram (Fig. 1). Inclusion was restricted to peer-reviewed journal articles, review articles, mixed-method studies, and survey reports focusing on prehospital or out-of-hospital emergency care system challenges in LMICs, classified according to World Bank economic criteria.

A total of 17 full-length articles were selected for the systematic review. Only studies published up to 2023 were included to maintain methodological rigor and reproducibility.

### 2.4 Assessment of risk of bias

The Critical Appraisal Skills Program (CASP) checklist was used as a tool to check for the validity and relevance of selected articles. It included 10 questions, which were scored with yes or no or cannot tell (Long et al., 2020).

### 2.5 Data analysis

We conducted a thematic analysis following the approach described by Braun and Clarke (2006) to synthesize findings across the included studies. An initial coding framework was developed deductively based on the World Health Organization (WHO) health systems building blocks framework, including service delivery, health workforce, health information systems, access to essential medicines, financing, and governance. This framework guided the identification of system-level barriers and integration challenges. All included studies were imported into NVivo- 14, where codes and nodes were systematically developed and organized. Two reviewers (KMY and AP) independently coded a subset of studies to ensure consistency in code application. The remaining studies were then coded, and emerging patterns were discussed collaboratively.

Additional codes and subthemes not captured within the initial framework were identified inductively from the data. Any discrepancies in coding or interpretation were resolved through discussion and consensus between the reviewers. Themes were refined iteratively to ensure they accurately reflected patterns and systemic barriers identified across the included studies.

### 2.6 Disclosure of ethical compliance

This study is based on a systematic review of previously published and publicly available data and does not constitute human subjects research because the study was based exclusively on previously published and publicly available data. Therefore, ethical approval was not required. The study adhered to standard ethical guidelines for the use of secondary data.

## 3 Results

### 3.1 Quality assessment

Based on the CASP appraisal, most included studies were of moderate to high methodological quality (Table 2). However, reporting and adjustment for confounding variables were inconsistent across studies. Despite these limitations, the studies provide valuable insights into EMS system performance and barriers in LMIC settings.

**Table 2:** Critical Appraisal Skills Program (CASP) quality assessment of 17 studies evaluating emergency medical service systems in low- and middle-income countries published between 2009 and 2023 [S1–S17].

S.N.	Authors (Year)	Country	Clear Focus	Appropriate Method	Clear Sample	Min Bias	Confounders Listed	Confounders Addressed	Complete Follow-up	CASP Quality
1	Alinia et al. (2015)	Iran	Y	Y	Y	CT	N	N	CT	Moderate
2	Bahadori & Ravangard (2013)	Iran	Y	Y	CT	CT	N	N	N/A	Moderate
3	Bhandari & Yadav (2020)	Nepal	Y	CT	CT	CT	N	N	CT	Low
4	Bhattarai et al. (2023)	LMICs	Y	Y	CT	Y	Y	CT	CT	High
5	Coyle & Harrison (2015)	Sierra Leone	CT	CT	CT	N	N	N	CT	Low
6	Kironji et al. (2018)	LMICs	Y	Y	CT	Y	Y	CT	CT	High
7	Mohd Said et al. (2014)	Malaysia	Y	Y	Y	CT	N	N	CT	Moderate
8	Nielsen et al. (2012)	13 LMICs	Y	Y	CT	CT	N	N	CT	Moderate
9	Niyonsaba et al. (2023)	Rwanda	Y	Y	Y	CT	N	N	CT	Moderate
10	Oliwa et al. (2023)	Kenya	Y	Y	Y	CT	N	CT	CT	Moderate
11	Roy et al. (2010)	India	Y	Y	Y	CT	N	N	CT	Moderate
12	Solagberu et al. (2009)	Nigeria	Y	Y	Y	CT	N	N	CT	Moderate
13	Sultan et al. (2019)	Ethiopia	Y	Y	Y	CT	N	N	CT	Moderate
14	Suriyawongpaisal et al. (2014)	Thailand	Y	Y	CT	CT	CT	CT	CT	Moderate
15	Sriram et al. (2016)	India/Pakistan	Y	Y	Y	CT	N	N	CT	Moderate
16	Suryanto et al. (2017)	LMICs	Y	Y	CT	CT	CT	CT	CT	Moderate
17	Wimalaratne et al. (2017)	Sri Lanka	Y	Y	CT	CT	CT	CT	CT	Moderate

Y = yes; N = No; CT = can't tell.

### 3.2 Regional variations in EMS systems

Substantial regional heterogeneity in EMS systems across LMICs was observed. The included studies were predominantly conducted in Sub-Saharan Africa, South and Southeast Asia, and the Middle East [S1–17]. The EMS system in Sub-Saharan African countries such as Ethiopia, Sierra Leone, Kenya, Nigeria, and Rwanda exhibited highly fragmented EMS systems hampered by limited funding, workforce shortages, and weak infrastructure [S1, S3, S5, S13, S15].

In South Asia, countries such as India and Pakistan demonstrated partially structured EMS systems [S8], while Nepal and Sri Lanka exhibited pronounced disparities between urban and rural service provision [S4, S9]. The level of EMS development varies widely across the region, with some countries having more established systems and others remaining in early stages of implementation (Oleribe et al., 2019). Similarly, Southeast Asia presented contrasting models of EMS organization. Thailand has developed a relatively integrated system combining public, private, and volunteer sectors under national coordination (Pochaisan et al., 2021), whereas Myanmar's EMS system remains largely informal and reliant on non-governmental and volunteer-based support due to the absence of a unified national framework (Bogale et al., 2024). These findings emphasize the context-specific nature of EMS development and highlight the challenges of generalizing across diverse LMIC settings.

### 3.3 Key EMS barriers and systems components

An overview of the included studies is summarized in Table 3. Deficiencies in ambulance infrastructure emerged as the most frequently reported barrier, encompassing limited ambulance availability, inadequate onboard equipment and medications, delayed response times, and failure to meet quality standards. Communication and coordination barriers were also prominent, with many studies reporting the absence of centralized dispatch systems, lack of standardized emergency contact numbers, and delays in information exchange between dispatch centers, patients, and ambulance teams.

**Table 3:** Summary of barriers and recommended strategies for strengthening emergency medical service systems in low- and middle-income countries based on studies published between 2009 and 2023.

Barrier type	Frequency (%)	Gaps and challenges
Funding	35% (6/17)	• Lack of proper funding for the EMS system • Inconsistent fund supplies for the regulation of emergency management (national level). • High out-of-pocket expenditure to utilize emergency services by patients (individual level).
Ambulance system	47% (8/17)	• Insufficient ambulance facilities in all parts • Low utilization of emergency vehicles • Lack of drugs and equipment inside the ambulance • Countries do not meet the international criteria for timely response and quality of ambulance services. • Response time is slower than the standard criteria.
EMS workforce	35% (6/17)	• Lack of Advanced Life Support training • Lack of standard accreditation of EMTs • Lack of paramedics and EMTs readily available during prehospital transportation • Insufficient dispatch officers/ team
Guidelines/policies	29% (5/17)	• Absence of a formal EMS structure, especially a prehospital care system • No specific models and/or guidelines • Problems in documentation with real-time communication. • Lacks a portal for data sharing, patient records, etc. • Guideline on a systematic way to transfer patients to appropriate health facilities
Communication	41% (7/17)	• Fewer number of dispatch centers • Lack of centralized communication centers • Lack of a uniform emergency number • Poor software quality • Risk of data breach • Problem in handling the calls at the dispatch centers • Delay in communication with the patient/ their family, and the ambulance team.
Accessibility/indicators	35% (6/17)	• Not accessible to the public • A toll-free number cannot be accessed by all • Coverage only in urban cities and specified areas. • Insufficient infrastructure, communication devices/ equipment, proper dispatch system leading to poor access to the public.
Public involvement	24% (4/17)	• Low awareness level • Lack of trust in public ambulance services • Low utilization and satisfaction rate for emergency services • Coordinating with patients/ family members at the accident scene

Financial constraints were another recurring theme, with insufficient and inconsistent funding contributing to gaps in service delivery and imposing high out-of-pocket costs on patients. Workforce-related issues were equally significant, including shortages of trained personnel, a lack of standardized certification for emergency medical technicians, and limited access to advanced life support training. Accessibility issues were frequently reported, particularly in rural and remote areas where poor infrastructure and limited-service coverage restrict timely access to emergency care.

In addition, weaknesses in policy and governance frameworks were identified, including the absence of centralized EMS systems and inadequate regulatory structures. Low levels of public awareness and engagement further contributed to the underutilization of EMS services and mistrust among communities. Several studies also highlighted the substantial role of informal and community-based responders (e.g., taxi drivers, volunteers), which often operate outside formal EMS structures despite contributing significantly to prehospital care. Additionally, emerging EMS initiatives and incremental system improvements were reported, suggesting a gradual shift toward more structured and coordinated prehospital care in some LMIC settings.

## 4 Discussion

This review demonstrates substantial heterogeneity in EMS development across LMICs, reflecting differences in governance, health system capacity, and resource availability [S12]. Because there is no universal model for EMS development (Nakahara et al., 2019), adaptation to local resources and needs is the key to successful EMS systems. Traditional HIC models require robust infrastructure and funding that are impractical for many LMICs. One example that showed great potential is Thailand's integrated approach, which combines centralized policy with public, private, and NGO collaboration (Pochaisan et al., 2021). This framework successfully bridges national guidelines with local-level innovation, adapting to regional resources and contexts.

In recent decades, the global burden of emergencies has risen due to infectious disease outbreaks, natural disasters, industrial accidents, and conflicts (Topluoglu et al., 2023). These challenges disproportionately affect LMICs with weaker health systems infrastructure. Infectious diseases like COVID-19 and Ebola can rapidly overwhelm emergency care systems (Bardhan et al., 2023). Climate change amplifies the frequency and severity of disasters such as floods and wildfires (Rocha et al., 2022; Rentschler et al., 2022). Additionally, industrial hazards, including chemical spills and explosions, complicate response efforts in settings with limited regulatory oversight (Wood and Fabbri, 2019). These compounding threats demonstrate that EMS must be resilient and adaptable. The intersection between health emergencies and climate events further reinforces the critical importance of integrating EMS within broader disaster preparedness and environmental health frameworks.

In many LMICs, public awareness of toll-free ambulance services provided by the local governments is lacking [S3]. This challenge is worsened by poor infrastructure and long transit distances. Overcoming these barriers requires EMS education and community mobilization [S3]. However, systemic development also demands adequate funding, strong leadership, integrated system components, standardized response guidelines, comprehensive legal frameworks, and professional workforce training for emergency medical technicians (EMTs) [S1]. In many contexts, informal and community-based emergency response mechanisms serve as de facto EMS systems. Rather than viewing these grassroots structures as evidence of system failure, policymakers should leverage them as foundational platforms for developing formalized national EMS systems.

Financial constraints remain a persistent and critical barrier to EMS development in LMICs [S4, S6, S7]. Chronic underfunding leads to severe shortages of medical supplies, ambulances, dispatch centers, and trained personnel. Furthermore, high out-of-pocket expenses deter patients from utilizing the EMS system during crises [S4, S6, S7]. Addressing these barriers requires sustainable financing models, such as universal health coverage (UHC), to ensure affordable and accessible EMS. Although select LMICs successfully offer free basic services and state-funded transport [S4], the long-term viability of any EMS system hinges entirely on consistent, institutionalized funding.

Beyond domestic constraints, EMS development is increasingly influenced by shifting global health financing priorities. Recent reductions in development assistance for health, coupled with a reallocation of funding toward climate change mitigation, geopolitical security, and national priorities in high-income countries, have widened financing gaps in LMIC health sectors (MacPherson et al., 2025). This transition heavily impacts EMS networks, which are frequently underprioritized in national budgets and reliant on external aid. Paradoxically, these funding contractions may expose underlying inefficiencies and reinforce the need for more resilient, self-reliant, and efficiency-driven EMS models.

Communication and coordination challenges represent an equally critical bottleneck. Many LMICs lack toll-free emergency numbers and centralized dispatch systems, severely delaying the interface between field responders and receiving hospitals [S2, S6]. While establishing a computer-aided dispatch (CAD) system offers a proven pathway to optimize efficiency and reduce response times [S8], initial setup costs are often prohibitive [S9, S13], presenting a major hurdle for resource-constrained regions.

A shortage of skilled EMS personnel and the absence of standardized training programs further compromise the quality of EMS in LMICs. As structured education in basic and advanced life support is scarce, establishing nationally regulated training, certification, and clinical protocols is vital for workforce competency [S6]. In addition, training must also emphasize communication and teamwork to streamline field coordination (Nurumal et al., 2014). Notably, this indicates the capacity–utilization paradox, whereby investments in training alone do not necessarily translate into improved system performance. In the absence of adequate infrastructure, equipment, and system integration, newly trained personnel may be underutilized or unable to apply their skills effectively. This structural bottleneck underscores the need for synchronized investments across workforce, infrastructure, and governance domains.

Fragmented governance, outdated legislation, and weak leadership are other critical barriers for EMS development in LMICs [S2, S7, S17]. The centralized and functional EMS systems require strong leadership from the government. Evidence-based policymaking, health system evaluation, and resource mapping can be performed to reform and identify investment priorities (Mehmood et al., 2018). In addition, expanding first-responder training to police officers, firefighters, taxi drivers, and community volunteers can help bridge human resource gaps, particularly in rural areas [S3, S10]. These findings suggest that EMS performance is not merely a reflection of clinical capacity but a broader indicator of governance effectiveness. Weak leadership, fragmented authority, and a lack of regulatory clarity undermine system-wide coordination, highlighting governance reform as a central component of EMS strengthening strategies.

A strong multisectoral partnership is also required to meet the operational and financial demands of EMS in LMICs. Collaboration with non-governmental organizations (NGOs) and private companies, and other community-based initiatives, plays a pivotal role in sharing the cost of infrastructure, manpower, and resources [S1, S13]. This can ensure that important emergency services remain accessible and equitable for every individual, particularly vulnerable populations (Walker et al., 2014). In many LMICs, these partnerships are not merely supplementary but constitute core components of EMS delivery. This challenges conventional assumptions that formal, state-led systems are the only viable model and highlights the potential of hybrid and collaborative approaches in resource-constrained settings.

Finally, public awareness remains a critical determinant of EMS utilization. Many people in LMICs are unaware of toll-free ambulance systems or government-supported services, even if they exist [S5, S7, S9]. Therefore, comprehensive EMS education is needed to effectively optimize system use. Table 4 brings these insights into sharper focus by pairing each major barrier with concrete, feasible strategies drawn from the evidence. With proper coordination, sustained investment, and community engagement, LMICs can establish locally adapted and resilient EMS systems that can strengthen the overall health systems of the country.

**Table 4:** Summary of barriers and recommended strategies for emergency medical services (EMS) in low- and middle-income countries.

Barrier category	Key issues identified	Recommended strategies
Governance & leadership	Weak policy frameworks, lack of coordination	Strengthen national EMS policies, improve governance structures, and enhance intersectoral coordination.
Funding	Limited funding, high out-of-pocket costs	Increase public investment, develop sustainable financing models, and integrate EMS into universal health coverage.
EMS workforce	Shortage of trained personnel, uneven distribution	Expand training programs, support task-shifting, and strengthen workforce retention strategies
Communication system	Poor dispatch systems, lack of centralized coordination	Develop integrated communication platforms, improve dispatch and referral systems.
Service delivery	Fragmented prehospital and hospital care	Improve integration between prehospital and facility-based services, standardize protocols.
Public involvement	Reliance on informal systems, low awareness	Strengthen community-based EMS models, increase public awareness, and provide first responder training.

Despite these potential pathways for improvement, several limitations should be acknowledged. First, the number of included articles was limited to those published from 2009 to 2023, which may have excluded other relevant studies. Secondly, only English-language, peer-reviewed articles that had access to full texts were selected, potentially excluding important research published in other languages or behind paywalls. Lastly, the inclusion criteria for the EMS system were broad, which didn't specifically focus on the EMS performance during disasters and other mass casualty events. Nevertheless, the findings provide valuable insights into systemic barriers and opportunities for strengthening more integrated and effective EMS systems in LMICs.

Overall, this review provides a comprehensive synthesis of EMS systems in LMICs. Importantly, it advances current understanding by demonstrating that EMS challenges are not solely resource-driven but are deeply rooted in system design, governance, and integration failures. Addressing these structural determinants is essential for developing resilient, equitable, and context-responsive EMS systems capable of responding to both routine emergencies and complex global health threats.

## 5 Conclusions

This systematic review found that emergency medical services in low- and middle-income countries remain variably developed, ranging from fragmented informal systems to partially integrated national structures. The principal barriers to effective EMS integration include weak governance, limited financing, workforce shortages, and poor coordination across health system components. Strengthening EMS in these settings requires context-specific, systems-oriented strategies that prioritize integration, governance, and multisectoral collaboration to support more equitable and resilient emergency care.

## CRediT authorship contribution statement

**Alina Pant:** Writing – original draft, Formal analysis, Writing – review & editing, Visualization. **Thunwadee Tachapattaworakul Suksaroj:** Conceptualization, Writing – review & editing. **Cheerawit Rattanapan:** Writing – review & editing. **Orapin Laosee:** Writing – review & editing. **Piyapong Janmaimool:** Writing – review & editing. **Kwang Mo Yang:** Writing – review & editing, Writing – original draft, Formal analysis, Data curation, Conceptualization.

## Funding

This research received no external funding.

## Declaration of competing interest

The authors declare that they have no known competing financial interests or personal relationships that could have appeared to influence the work reported in this paper.

## Data Availability

Data will be made available on request.

## Appendix A Supplementary data

Supplementary material References for studies included in the systematic review
