# Supplementary material for: Emergency medical services in low- and middle-income countries: a systematic review of systemic barriers and integration challenges
Source: Prev Med Rep. 2026 Jun 3;67:103522. doi: 10.1016/j.pmedr.2026.103522 (PMC13254714; doi:10.1016/j.pmedr.2026.103522)
Supplement: Supplementary material — References for studies included in the systematic review [file mmc1.docx]

**Supplementary file 1. References for studies included in the systematic review**

**Supplementary References**

S1 Nielsen K, Mock C, Joshipura M, Rubiano AM, Zakariah A, Rivara F. Assessment of the status of prehospital care in 13 low- and middle-income countries. Prehosp Emerg Care. 2012;16:381-9.

S2 Kironji AG, Hodkinson P, de Ramirez SS, Anest T, Wallis L, Razzak J, et al. Identifying barriers for out of hospital emergency care in low and low-middle income countries: a systematic review. BMC Health Serv Res. 2018;18:291.

S3 Sultan M, Abebe Y, Tsadik AW, Ababa A, Yesus AG, Mould-Millman N-K. Trends and barriers of emergency medical service use in Addis Ababa; Ethiopia. BMC Emergency Medicine. 2019;19:28.

S4 Bhandari D, Yadav NK. Developing an integrated emergency medical services in a low-income country like Nepal: a concept paper. International Journal of Emergency Medicine. 2020;13:7.

S5 Coyle RM, Harrison H-L. Emergency care capacity in Freetown, Sierra Leone: a service evaluation. BMC Emergency Medicine. 2015;15:2.

S6 Suryanto, Plummer V, Boyle M. EMS systems in lower-middle income countries: A literature review. Prehospital and Disaster Medicine. 2017;32:64-70.

S7 Roy N, Murlidhar V, Chowdhury R, Patil SB, Supe PA, Vaishnav PD, et al. Where there are no emergency medical services-prehospital care for the injured in Mumbai, India. Prehosp Disaster Med. 2010;25:145-51.

S8 Sriram V, Gururaj G, Razzak JA, Naseer R, Hyder AA. Comparative analysis of three prehospital emergency medical services organizations in India and Pakistan. Public Health. 2016;137:169-75.

S9 Wimalaratne K, Lee JI, Lee KH, Lee HY, Lee JH, Kang IH. Emergency medical service systems in Sri Lanka: problems of the past, challenges of the future. Int J Emerg Med. 2017;10:10.

S10 Solagberu BA, Ofoegbu CK, Abdur-Rahman LO, Adekanye AO, Udoffa US, Taiwo J. Pre-hospital care in Nigeria: a country without emergency medical services. Niger J Clin Pract. 2009;12:29-33.

S11 Mohd Said N, Sukonthasarn A, Wangsrikhun S, Chanpransit C. Assessing and exploring the competency of prehospital emergency medical service personnel in Klang Valley, Malaysia: A mixed method approach. International Medical Journal Malaysia. 2014;13:7-20.

S12 Bhattarai HK, Bhusal S, Barone-Adesi F, Hubloue I. Prehospital emergency care in low- and middle-income countries: A systematic review. Prehospital and Disaster Medicine. 2023;38:495-512.

S13 Oliwa JN, Mazhar RJ, Serem G, Khalid K, Amoth P, Kiarie H, et al. Policies and resources for strengthening of emergency and critical care services in the context of the global COVID-19 pandemic in Kenya. PLOS Glob Public Health. 2023;3:e0000483.

S14 Bahadori M, Ravangard R. Determining and Prioritizing the Organizational Determinants of Emergency Medical Services (EMS) in Iran. Iran Red Crescent Med J. 2013;15:307-11.

S15 Niyonsaba M, Nkeshimana M, Uwitonze JM, Davies J, Maine R, Nyinawankusi JDA, et al. Challenges and opportunities to improve efficiency and quality of prehospital emergency care using an mHealth platform: Qualitative study in Rwanda. African Journal of Emergency Medicine. 2023;13:250-7.

S16 Alinia S, Khankeh H, Maddah SS, Negarandeh R. Barriers of pre-hospital services in road traffic injuries in Tehran: The viewpoint of service providers. Int J Community Based Nurs Midwifery. 2015;3:272-82.

S17 Suriyawongpaisal P, Aekplakorn W, Tansirisithikul R. A Thailand case study based on quantitative assessment: does a national lead agency make a difference in pre-hospital care development in middle income countries? Scand J Trauma Resusc Emerg Med. 2014;22:75.
